# Boosting Serotonin Increases Information Gathering by Reducing Subjective Cognitive Costs

**DOI:** 10.1523/JNEUROSCI.1416-22.2023

**Published:** 2023-08-09

**Authors:** Jochen Michely, Ingrid M. Martin, Raymond J. Dolan, Tobias U. Hauser

**Affiliations:** ^1^Department of Psychiatry and Neurosciences, Charité–Universitätsmedizin Berlin, Corporate Member of Freie Universität Berlin and Humboldt-Universität zu Berlin, Berlin, 10117 Germany; ^2^Berlin Institute of Health at Charité–Universitätsmedizin Berlin, BIH Charité Clinician Scientist Program, Berlin, 10117 Germany; ^3^Max Planck UCL Centre for Computational Psychiatry and Ageing Research, University College London, London, WC1B 5EH, United Kingdom; ^4^Wellcome Centre for Human Neuroimaging, University College London, London, WC1N 3BG, United Kingdom; ^5^Institute of Cognitive Neuroscience, University College London, London, WC1N 3AZ, United Kingdom; ^6^Department of Psychiatry and Psychotherapy, Medical School and University Hospital, Eberhard Karls University of Tübingen, 72076 Tübingen, Germany; ^7^German Center for Mental Health (DZPG)

**Keywords:** cognitive effort, computational modeling, information gathering, serotonin

## Abstract

Serotonin is implicated in the valuation of aversive costs, such as delay or physical effort. However, its role in governing sensitivity to cognitive effort, for example, deliberation costs during information gathering, is unclear. We show that treatment with a serotonergic antidepressant in healthy human individuals of either sex enhances a willingness to gather information when trying to maximize reward. Using computational modeling, we show this arises from a diminished sensitivity to subjective deliberation costs during the sampling process. This result is consistent with the notion that serotonin alleviates sensitivity to aversive costs in a domain-general fashion, with implications for its potential contribution to a positive impact on motivational deficits in psychiatric disorders.

**SIGNIFICANCE STATEMENT** Gathering information about the world is essential for successfully navigating it. However, sampling information is costly, and we need to balance between gathering too little and too much information. The neurocomputational mechanisms underlying this arbitration between a putative gain, such as reward, and the associated costs, such as allocation of cognitive resources, remain unclear. In this study, we show that week-long daily treatment with a serotonergic antidepressant enhances a willingness to gather information when trying to maximize reward. Computational modeling indicates this arises from a reduced perception of aversive costs, rendering information gathering less cognitively effortful. This finding points to a candidate mechanism by which serotonergic treatment might help alleviate motivational deficits in a range of mental illnesses.

## Introduction

Harvesting information about the world is essential for successfully navigating it. However, gathering information is costly and we need to balance between gathering too little and too much information (Gottlieb et al., 2013). Excess information gathering for a simple or irrelevant decision, such as repetitively checking whether the door is lock, or the oven switched off, may lead to unnecessary costs, including the time and energy spent exploring, such as in patients suffering from obsessive-compulsive disorder (OCD; Hauser et al., 2017a; Strauss et al., 2020). Alternatively, expending too little resources on information gathering, e.g., because of an aversion to allocate cognitive resources in patients suffering from depression, may increase the risk of jumping to conclusions, resulting in unwarranted assumptions and poor decisions (Taylor Tavares et al., 2007; Sastre-Buades et al., 2021).

This challenge in information gathering can be reformulated as an arbitration between the value of novel information and the cognitive costs incurred. Humans struggle with solving this arbitration optimally, often showing excessive or insufficient information gathering (Bogacz et al., 2010; Hauser et al., 2017b). Interestingly, suboptimalities in information gathering are a feature in a range of psychiatric disorders (Clark et al., 2006; Taylor Tavares et al., 2007; Moutoussis et al., 2011; Hauser et al., 2017a, b; Ermakova et al., 2019).

The neurocomputational mechanisms underlying this arbitration between a putative gain, such as reward, and the associated costs, such as allocation of cognitive resources, remain unclear. It is hypothesized that the neurotransmitter serotonin may play a role in this process (Husain and Roiser, 2018). Although the exact impact of serotonin on decision-making remains somewhat elusive (Cools et al., 2011; Dayan, 2012), it has been suggested as signaling a cost related to action, such as physical effort (Meyniel et al., 2016) or action inhibition (Crockett et al., 2009; Guitart-Masip et al., 2014).

Meyniel et al. (2016) demonstrated that boosting serotonin by selective serotonin reuptake inhibitors (SSRIs) reduces a perception of physical effort by lowering a sensitivity to its associated aversive costs. Likewise, it has been hypothesized that serotonin's impact on intertemporal choice may reflect a reduced cost perception for delay-induced costs (Schweighofer et al., 2008; Miyazaki et al., 2014; Fonseca et al., 2015). However, it remains uncertain whether serotonin signals a cost beyond mere physical effort, for example, the cost of cognitive effort critical for deliberation and information gathering (Froböse and Cools, 2018; Petitet et al., 2021).

In this study, we tested whether serotonin alleviates cognitive cost sensitivity in information gathering. Using a double-blind, placebo-controlled, between-subjects design, we assessed the impact of week-long daily treatment with the SSRI citalopram on decision-making during an established sequential information gathering task (Hauser et al., 2017a,b). We find SSRI treatment boosts information gathering in a manner indicative of subjects being more willing to exert cognitive effort to obtain reward. Computational modeling revealed this serotonergic effect is specifically driven by a reduction in the aversive cost of deliberation. The findings are consistent with serotonin playing a domain-general role in encoding ongoing both physical and cognitive costs, a mechanism that might be relevant for therapeutic approaches to motivational deficits in psychiatric disorders.

## Materials and Methods

### Subjects

A total of 66 healthy volunteers (age range 20–38 years; SSRI, 20 females, 13 males, mean age: 24.5 ± 4.0; placebo, 20 females, 13 males, mean age: 24.8 ± 3.9, *p* = 0.757) participated in this double-blind, placebo-controlled study. All subjects underwent an electrocardiogram to exclude QT interval prolongation and a thorough medical screening interview to exclude any neurologic or psychiatric disorder, any other medical condition, or medication intake. The experimental protocol was approved by the University College London (UCL) local research ethics committee, with informed consent obtained from all participants. Data from different tasks of the same participants were already published elsewhere (Michely et al., 2020, 2022).

### Pharmacological procedure

Participants were randomly allocated to receive a daily oral dose of the SSRI citalopram (20 mg) or placebo, over a period of 7 consecutive days. All subjects performed two laboratory testing sessions. The first session was on day 1 of treatment, approximately 3 h after single dose administration, as citalopram reaches its highest plasma levels after this interval (Noble and Benfield, 1997). On the following days, subjects were asked to take their daily medication dose at a similar time of day, either at home or at the study location. The second session was on day 7 of treatment, with the tablet being taken approximately 3 h before the experiment.

### Experimental task

**Figure 1.**
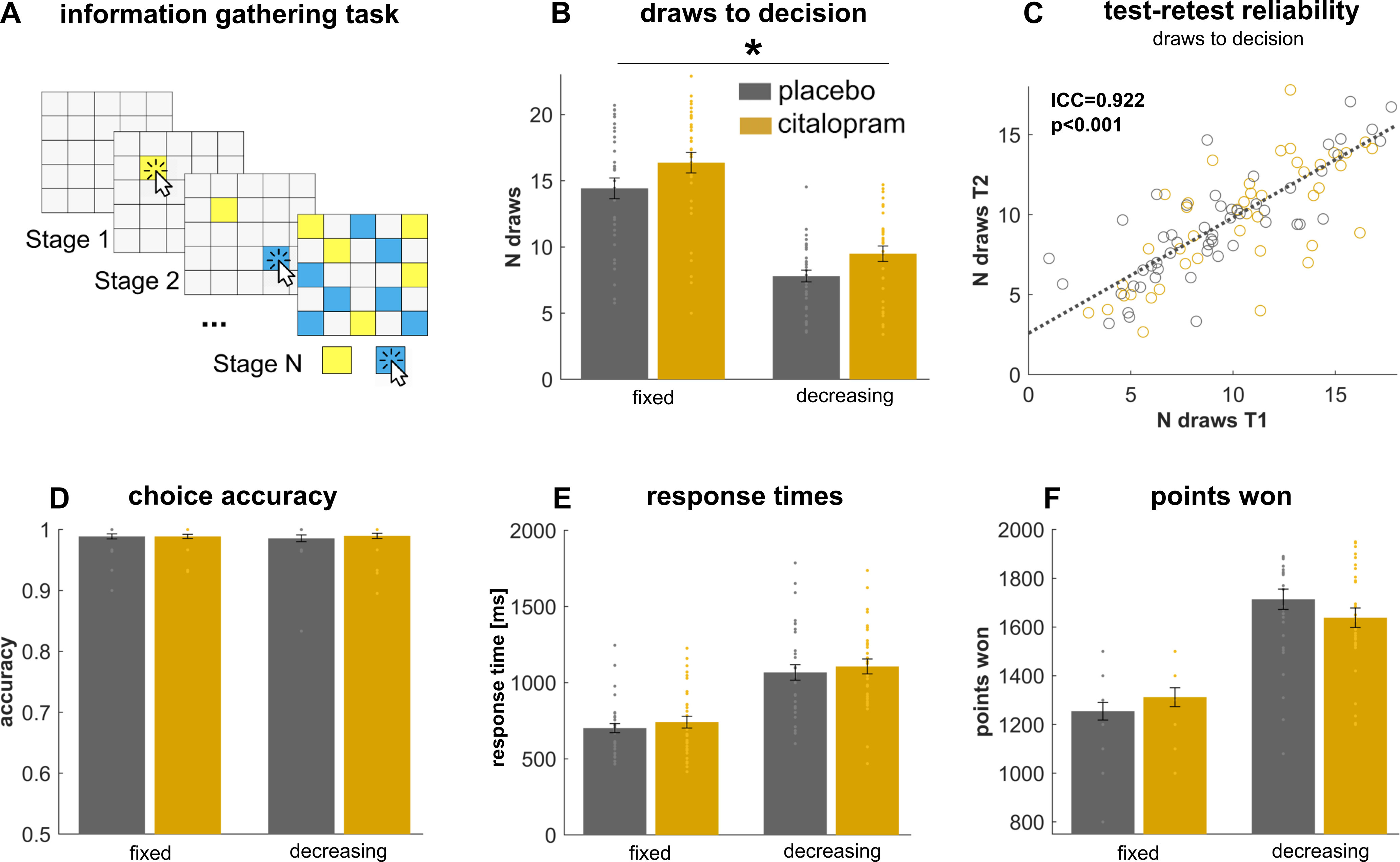
Increased information gathering after SSRI administration. ***A***, Subjects were randomly allocated to a daily dose of 20-mg citalopram or placebo for 7 consecutive days. Subjects performed the information gathering task on two sessions: session 1 took place on day 1 after a single-dose administration, session 2 took place on day 7 after a week-long drug treatment. For each game, subjects started with a fully covered deck of 25 gray cards. They were allowed to open as many cards as they wished before declaring a decision about whether they believed the majority of cards was yellow or blue (colors varied across games). In the “fixed” condition, no external costs for sampling applied, but in a “decreasing” condition a potential total win of 250 points reduced by 10 points per uncovered card. In both conditions, subjects lost 100 points on making an incorrect decision. ***B***, SSRIs led to a general increase in the number of draws needed before declaring a decision (shown here collapsed across both timepoints as there were no interactions with time). This was evident across both conditions. ***C***, Information gathering (number of draws) shows a high test-retest reliability, demonstrating that the task is capable of reliably detecting individual differences in information gathering. ***D–F***, There were no significant drug effects on further task metrics, such as choice accuracy, response times or task earnings. *p < 0.028. Error bars indicate SEM. See Extended Data Figure 1-1 for self-report questionnaire data. ms = milliseconds, N draws = number of draws.

We examined sequential information gathering using a modified version of an information sampling task (Clark et al., 2006; Hauser et al., 2017a,b). On each game, subjects saw 25 covered cards (Fig. 1*A*, gray squares) and had to decide whether the majority of cards was of color 1 or color 2 (e.g., yellow or blue, colors varied across games). Using a computer mouse, subjects were allowed to sample as many cards as they wished before committing to one of the two colors.

The first 15 games were part of a “fixed” condition, in which gathering additional information was not costly. Specifically, subjects received 100 points for correct decisions and lost 100 points for incorrect decisions, regardless of the number of cards opened or the time spent on task before decision. In the “decreasing” condition, information gathering incurred external costs resulting in a reduction of potential gains. Specifically, starting from a maximum potential gain of 250 points, opening each card led to a 10-point gain reduction (e.g., gain after seven opened cards: 250 − 7 × 10 = 180 points). Incorrect decisions resulted in a loss of 100 points, independent of the amount of prior sampling.

After each game, subjects were informed about their gains, and then proceeded to the next game. The game sequences were selected so that 10 games in each condition were relatively difficult with a generative probability close to 50% (similar to that in the original information sampling task; (Clark et al., 2006). An additional five sequences were easier with a clearer majority (generative probabilities of a binomial process *p* ∼ 0.7) to allow for a broader variability in information gathering. Order of sequences was randomized. Before the first game, subjects performed a practice game to familiarize themselves with the task.

### Computational modeling

To investigate the computational mechanisms underlying information gathering, we used a computational model that we have previously developed for this task. Here, we reiterate the most relevant equations, but a detailed description of the model can be found in a previously published work (Hauser et al., 2017b) and subsequent papers (Hauser et al., 2017a, 2018; Bowler et al., 2021).

The computational model assumes that agents make inference about which color is more likely to form the majority of cards $P(MY|n_{y},N)$ with *MY* being a majority of yellow, given the current amount of yellow cards (*n_y_*) out of a total of *N* sampled cards. *P(MY)* is calculated using the current number of cards, making inference about the generative probability that could have caused this distribution of cards (cf. Hauser et al., 2017b).

This belief is then used to calculate action for declaring for yellow and blue, weighting both potential gains and losses by the inferred likelihood of them taking place

$$Q(Y|n_{y},N)=R_{cor}P(MY|n_{y},N)+R_{inc}P(MB|n_{b},N),$$

with *R_cor_* and *R_inc_* being the potential wins and losses (set to +100/−100 here; for discussion, cf. Hauser et al., 2018).

A more challenging computation is the estimation of the action value for not deciding [*Q(ND)*]. This is computed as the sum of the value of the future states [*V(s')*, a weighted sum of the *Q* values in that state], weighted by how likely they will materialize [*p(s*'|*n_y_,N)*, i.e., how likely will I end up in that state given the cards that I have opened so far]. In addition, a cost per step *c* incurs, which accounts for the subjective costs for sampling more information and thus spending more effort, time, and points (in the decreasing condition) on gathering information.

$$Q\left(ND\text{|}n_{y},N\right)=\overset{i=\left[0,1\right]}{\underset{s\prime =\left\{\begin{array}{c} n_{y}+i\\ N+1 \end{array}\right\}}{\sum }}P\left(s^{\prime }\text{|}n_{y},N\right)V\left(s^{\prime }\right)+c$$

In accordance with previous studies, we found that subjective costs did not follow the explicit costs (i.e., 0 in the fixed, −10 points in the decreasing condition; “objective” model). Moreover, costs also did not increase linearly (“linear” model), but rather scaled in a nonlinear fashion during sampling (“nonlinear” model, modeled as a sigmoid). The best performing, nonlinear, model comprised two free parameters, a scaling parameter *cs* that determined how big the maximal costs could be, and an intercept *p*, which determined after how many samples (*n*) these costs started to escalate (Drugowitsch et al., 2012; Murphy et al., 2016).

$$c=\frac{cs}{1+e^{-10(n-p)}}$$

Amending our earlier work (Hauser et al., 2017a,b), our recent modeling analyses on data from the same task (Hauser et al., 2018; Bowler et al., 2021) revealed that fixing the slope parameter (*k*) to 10, instead of having it as additional free parameter, leads to similar model fit, showing that this parsimonious model thus outperforms more complex models. Fixing the slope of our cost function means that a potential effect on the slope would likely be reflected in the *cs* or *p* parameter. However, we have little reason to believe that serotonin would affect the slope exclusively, or even that slope and costs would be consequences of distinct neurocognitive processes.

Lastly, the choice policy was determined using a softmax rule with decision temperature τ and an additional ε greedy element (ξ) that captures choices that were not adequately captured by the model. The policy was not only used for choice arbitration, but also in the planning process to inform state values und backward planning (for details, cf. Hauser et al., 2017b):

$$\pi (ND|n_{y},N)=\frac{e^{Q(ND|n_{y},N)/\tau }}{e^{Q(Y|n_{y},N)/\tau }+e^{Q(B|n_{y},N)/\tau }+e^{Q(ND|n_{y},N)/\tau }}(1-\xi )+\frac{\xi }{3}.$$

To determine the best-fitting model, we compared three distinct models with different forms of cost structures as described above: objective, linear, nonlinear. To compare their model fit, we applied out-of-sample prediction using a 5-fold cross-validation assessing the predictive likelihood, thereby finding an optimal balance between complexity and accuracy using the held-out data (Dubois et al., 2021). Specifically, we partitioned the data of each subject into five folds (subsamples). We then fitted the model using four folds and validated it on the remaining fold. We repeated this procedure five times so that each fold is used as a validation set once, and averaged the likelihood over held-out trials. We computed this for each model and subject and averaged across subjects. Model parameters were optimized using 'fmincon,' with multiple starting points to overcome local minima.

Thus, our methodological approach comprised two stages. First, we compared different types of models (objective, linear, nonlinear) to assess whether costs were represented as per explicit instruction or whether subjective costs accumulated in a linear, or nonlinear, fashion. Second, we assessed whether changes in information gathering, such as differences in the number of draws, can be mapped to distinct changes of certain parameters of the model, such as the sensitivity to sampling costs (*cs* parameter) or the indifference point that is governing after how many samples subjective costs start to escalate (*p* parameter).

Note that the main models included two τ and two *p* parameters (separate for each condition), and one *cs* parameter (shared across conditions), as our most recent work showed that such models provide the best fir for the data of this task (Hauser et al., 2017a, 2018; Bowler et al., 2021). Nevertheless, to further elucidate cost of sampling effects, we also computed an additional model that included two separate *cs* parameters, one per condition.

### Statistical analysis

In this study, we tested whether SSRI treatment affects information gathering. The number of draws before a decision is a good indicator for the amount of information that a subject is willing to collect before committing to a decision (Hauser et al., 2017a,b). We analyzed this behavioral metric using repeated-measures ANOVAs with the between-subject factor drug (SSRI, placebo), and the within-subject factor condition (fixed, decreasing). To assess whether effects were different after single-dose administration, compared with one-week treatment, we added the within-subject factor time (session 1: acute; session 2: week-long). Significant effects were further assessed using independent-sample *t* tests (SSRI vs placebo). As secondary measures (Hauser et al., 2017a,b), we assessed whether drug treatment affected how many points subjects won, and how accurate subjects were in their decision-making (i.e., how often subjects correctly opted for the color with the current majority of cards), using the same statistical procedures. For comparison of computational model parameters, we applied Bonferroni correction for the number of model parameters.

## Results

### Serotonin increases information gathering

First, we assessed the number of draws a subject made before committing to a decision as a key indicator of information gathering in the task. This metric is a proxy for a subject's need to gather information, and has been found sensitive to individual differences including levels of psychopathology (Clark et al., 2006; Taylor Tavares et al., 2007; Moutoussis et al., 2011; Hauser et al., 2017a,b).

Here, we found a significant main effect of drug (*F*_(1,64)_ = 5.0, *p* = 0.028; Fig. 1*B*), driven by an increase in number of draws in the SSRI compared with placebo subjects. Additionally, we found a significant effect of condition (*F*_(1,64)_ = 202.3, *p* < 0.001), indicating that subjects gathered more information in the fixed compared with the decreasing condition. However, we found no interaction between drug and condition (*F*_(1,64)_ = 0.07, *p* = 0.793), indicating that SSRIs increased information gathering per se, and drug effects were similar across conditions (SSRI vs placebo: decreasing: *t*_(64)_ = 2.3, *p* = 0.025; fixed: *t*_(64)_ = 1.8, *p* = 0.084). Further, the duration of drug administration (single-dose vs week-long) did not impact the drug's effect on information gathering (drug × time: *F*_(1,64)_ = 0.9, *p* = 0.345), or the interaction with condition (drug × time × condition: *F*_(1,64)_ = 0.3, *p* = 0.602). This means that the drug effects were similar for acute and prolonged treatment. Overall, these results show that (acute and week-long) serotonergic treatment enhanced a willingness to gather more information before declaring a choice.

10.1523/JNEUROSCI.1416-22.2023.f1-1 Extended Data Figure 1-1 Self-report questionnaire data. We found no impact of drug on any of self-report questionnaires. BDI–II = Beck's Depression Inventory II (Beck et al., 1996); SHAPS = Snaith-Hamilton Pleasure Scale (Snaith et al., 1995); STAI = State-Trait Anxiety Inventory (Spielberger, 1983); PANAS = Positive and Negative Affective Scale (Watson et al., 1988). Download Figure 1-1, DOCX file.

### Serotonergic effects on further task metrics

Next, we assessed whether SSRIs affected other metrics, not directly related to information gathering. First, we examined subjects' accuracy, as measured by whether a subject chooses the color that is more plentiful at the time of decision. We did not find any pharmacological effect on accuracy, suggesting that SSRIs do not simply change motivation, attention or information processing in general (drug: *F*_(1,64)_ = 0.1, *p* = 0.711; drug × time: *F*_(1,64)_ = 0.7, *p* = 0.402; drug × condition: *F*_(1,64)_ = 0.4, *p* = 0.537; drug × time × condition: *F*_(1,64)_ = 0.5, *p* = 0.482; Fig. 1*D*).

Next, we analyzed choice response times. As expected, we found that subjects responded faster in the fixed as compared with the decreasing condition (*F*_(1,64)_ = 151.3, *p* < 0.001). However, there was no pharmacological effect on response times (drug: *F*_(1,64)_ = 0.6, *p* = 0.457, drug × condition: *F*_(1,64)_ = 0.0, *p* = 0.99; drug × time: *F*_(1,64)_ = 1.7, *p* = 0.202; drug × time × condition: *F*_(1,64)_ = 0.5, *p* = 0.481; Fig. 1*E*).

Lastly, we investigated pharmacological effects on task earnings, i.e., how many points subjects won in the task. This measure is a conglomerate measure influenced by information gathering, accuracy, and luck. Note that an SSRI-induced increase in sampling across both conditions would be reflected in an increase in earnings in the fixed condition (where sampling is cost-free) and a reduction in earnings in the decreasing condition (where sampling is costly; Hauser et al., 2017b). Note, however, this drug by condition interaction did not reach statistical significance (*F*_(1,64)_ = 3.6, *p* = 0.060). Further, we found this measure to be unaffected by treatment in the remaining comparisons (drug: *F*_(1,64)_ = 0.04, *p* = 0.834; drug × time: *F*_(1,64)_ = 0.01, *p* = 0.915; drug × time × condition: *F*_(1,64)_ = 0.5, *p* = 0.478; Fig. 1*F*).

### Emerging subjective costs reduce information gathering

To decipher the computational mechanisms that drive an increase in information gathering in SSRI-treated subjects, we fitted three different computational models to individual subjects' choices (see Materials and Methods). These previously developed and evaluated models cast information gathering as a trade-off between information gain of a new sample and the incurred costs in sampling information. In particular, they capture subjective information gathering costs in the context of Bayesian decision-making and characterize how these costs change as information gathering continues (Hauser et al., 2017a,b; Bowler et al., 2021).

**Figure 2.**
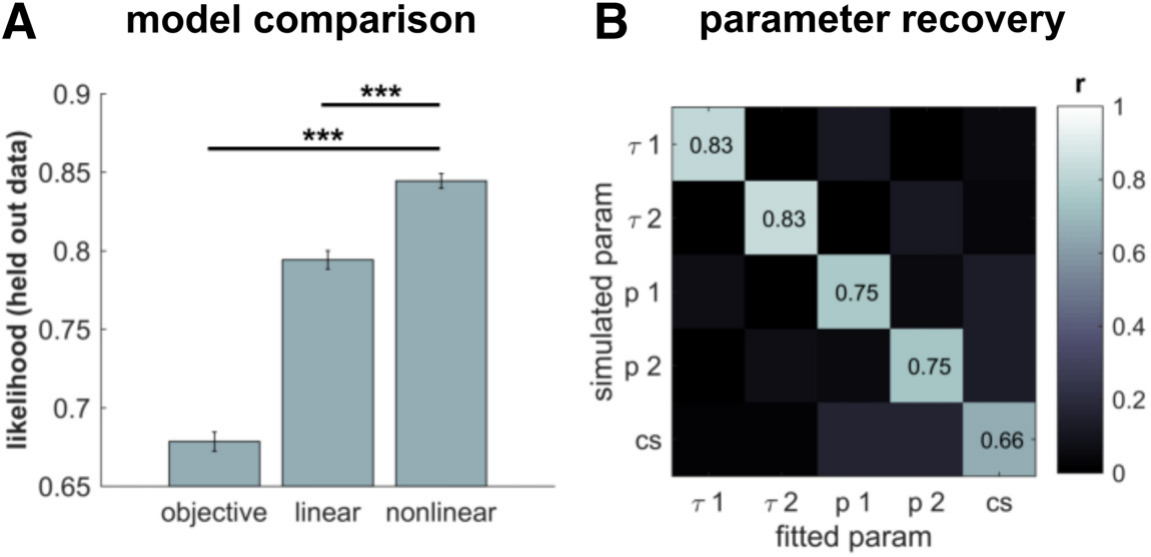
Computational modeling, model comparison and parameter recovery. ***A***, Model comparison predicting cross-validated hold-out data revealed that a nonlinear increase in subjective sampling costs fitted subjects' performance best. ***B***, Confusion matrices of the winning model shows that the parameters could be recovered using 50,000 simulated agents. This was demonstrated by medium to large correlations of the fitted with the original parameters used for simulation. Please note that the “xi” parameter was not included in the model simulations but was kept as a free parameter in the model fitting. This means a trade-off would be expressed in lowered correlation values for our parameters of interest. 1: fixed condition; 2: decreasing condition. ***p < 0.001. Error bars indicate SEM.

In line with our previous findings (Hauser et al., 2017a,b; Bowler et al., 2021), the winning model revealed that the cost for gathering information is subjective and changes over the course of information gathering. Model comparison revealed that these costs were not represented as per explicit instruction (“objective” model, i.e., no costs for the fixed, and −10 for the decreasing condition), but that “subjective” costs accumulated as information gathering continued, meaning that it becomes subjectively more and more costly to gather further information as a function of cumulative information gathering. This effect was best captured by a model in which costs escalate in a “nonlinear,” rather than a “linear,” fashion (“nonlinear” vs “objective”: *t*_(65)_ = 16.3, *p* < 0.001; “nonlinear” vs “linear”: *t*_(65)_ = 8.6, *p* < 0.001; Fig. 2*A*), in accordance with previous studies which identified the same winning model (Hauser et al., 2017a,b, 2018; Bowler et al., 2021).

When we investigated how serotonin impacted putative mechanisms underlying information gathering, using simulations, we found that model parameter estimates could be accurately recovered, demonstrated in a positive association between parameter estimates derived from fitting to real and simulated data (Fig. 2*B*).

### Serotonin decreases subjective costs of information gathering

**Figure 3.**
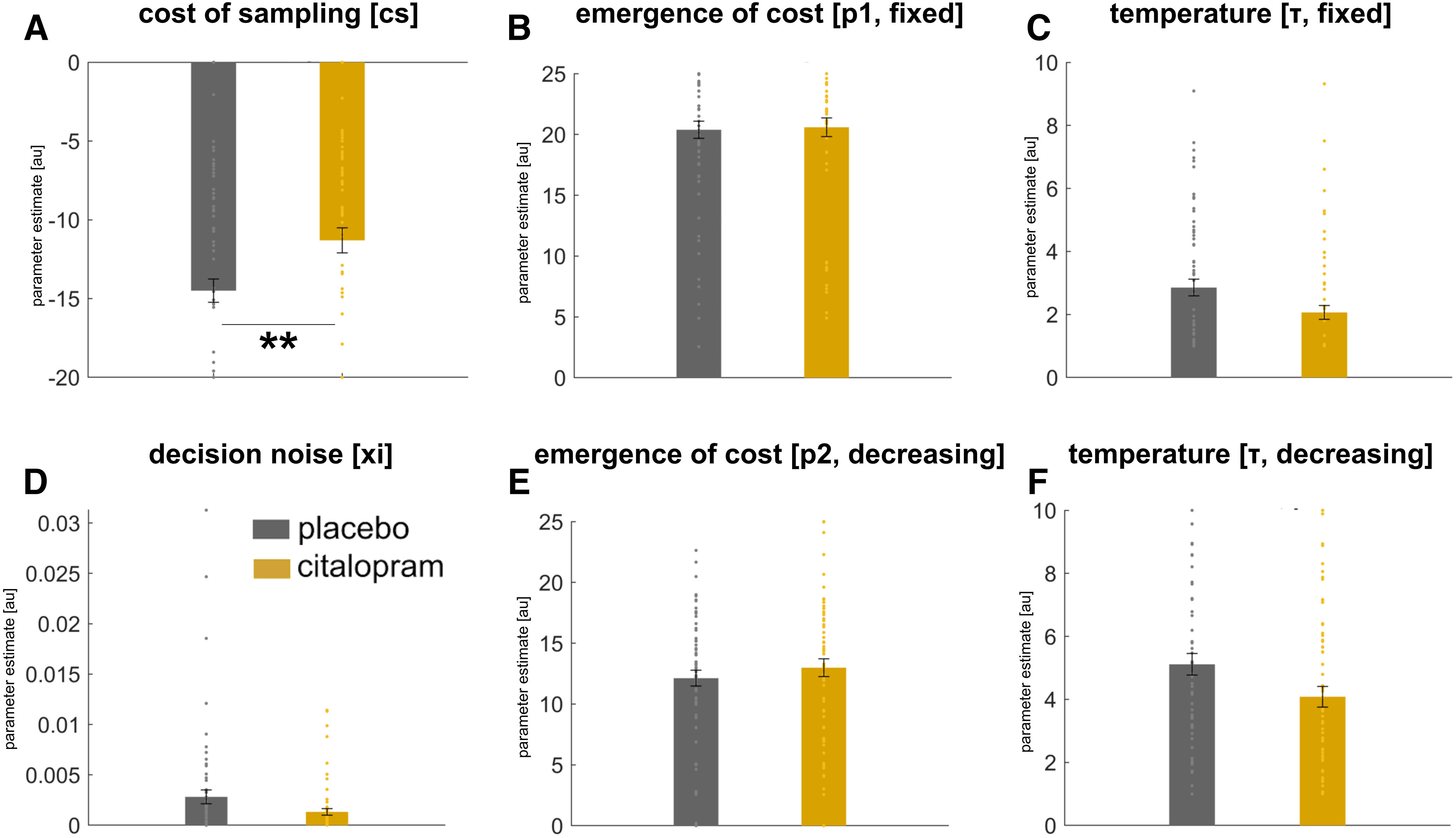
Computational modeling, parameter estimates. ***A***, SSRIs reduce subjective costs in information gathering, indicated by a lower subjective cost model parameter in citalopram as compared with placebo subjects (collapsed across both time points as there was no time effect on result). ***B–F***, We found no effect of drug on any of the other model parameters. **p = 0.007, uncorrected; *p* = 0.042, Bonferroni-corrected. Error bars indicate SEM. au = arbitrary units.

To better understand how serotonin impacted information gathering, we compared the model parameters between the two groups. We found only one model parameter that survived multiple comparison correction (Fig. 3). In particular, we found that SSRI treatment specifically affected the subjective cost parameter *cs* (*F*_(1,64)_ = 7.8, *p* = 0.007, uncorrected; *p* = 0.042, Bonferroni-corrected; Fig. 3*A*). This parameter is less negative in the SSRI group, which means that SSRI treatment led to a reduction in the subjective costs of information gathering. In alignment with the behavioral findings, there was no effect of time (acute vs week-long treatment) on this parameter (drug × time interaction: *F*_(1,64)_ = 0.056, *p* = 0.814), meaning that acute and week-long treatment had a similar effect. To elucidate the subjective effects of costs further, we computed an additional model that comprised two separate *cs* parameters, one per condition. This analysis revealed that parameter estimates were different between conditions (*F*_(1,64)_ = 9.9, *p* = 0.002), with higher cost sensitivity in the “decreasing” (costly) as compared with the “fixed” (not costly) condition. Further – in line with our results in the main analysis – we found a significant effect of “drug” (*F*_(1,64)_ = 4.9, *p* = 0.030). Additionally, there was a significant “drug” × “condition” interaction (*F*_(1,64)_ = 4.7, *p* = 0.033), an effect driven by a significantly higher cost sensitivity in SSRI as compared to placebo subjects in the “decreasing” (*t*_(64)_ = 2.9, *p* = 0.004), but not in the “fixed” condition (*t*_(64)_ = 0.3, *p* = 0.765). In sum, this indicates that an increase in information gathering in SSRI-treated subjects arises as a consequence of a lowered sensitivity to the subjective cost of sampling after serotonergic intervention, and this effect is particularly prominent in the decreasing condition when sampling information is costly.

### Test-retest reliability

A potential limitation with task-based behavioral metrics, as with many other cognitive variables (Shahar et al., 2019), is that little is known about their psychometric properties, such as test-retest reliability. Good psychometric properties are particularly important when tasks are used to make inference across repeated measurements, or when used to differentiate between subjects (e.g., patient vs nonpatient groups, drug vs no-drug groups). Given that we assessed the same subjects twice, with a 7-day interval between measurements, we were in the unique position to assess the test-retest reliability. Here, we found excellent reliability for our main measure of interest, the number of draws [intraclass correlation (ICC) = 0.922, *p* < 0.001; Fig. 1*C*]. This means that our information gathering task is well-suited for studying individual differences. For the computational modeling parameters, we found significant effects across all model parameters (all *p* < 0.012), however, coefficients were overall lower than our main model-free measure (ICCs ranging from 0.429 to 0.756). More sophisticated modeling approaches (Shahar et al., 2019) could help improve the psychometric properties of these measures further.

### No drug effects on self-report questionnaires

To examine putative treatment effects on subjective affective states over the course of the study, participants completed the Beck's Depression Inventory (BDI-II; Beck et al., 1996), Snaith-Hamilton Pleasure Scale (SHAPS; Snaith et al., 1995), State-Trait Anxiety Inventory (STAI; Spielberger et al., 1983), and the Positive and Negative Affective Scale (PANAS; Watson et al., 1988) on two different occasions: (1) predrug, day 1; (2) peak drug, day 7. We found no evidence for serotonergic effects on any of the self-report questionnaires (compare Extended Data Fig. 1-1). This is in line with previous studies showing week-long SSRI treatment does not impact on mood in healthy volunteer participants (Harmer, 2013).

## Discussion

We show that boosting serotonin function pharmacologically leads to increased information gathering in a sequential information sampling task. Using computational modeling, we demonstrate this effect arises from a serotonin-induced reduction in subjective sampling costs.

Serotonin is an impactful neuromodulator, but its precise influence on cognition, motivation and behavior remains elusive (Dayan, 2012; Olivier, 2015). While early accounts proposed serotonin as an opponent to dopamine that is mainly involved in signaling aversive outcomes (Daw et al., 2002; Cools et al., 2011), recent evidence has drawn a more complex and nuanced picture. In particular, studies suggest serotonin plays a crucial role in signaling the costs that are associated with aversive experience. These costs encompass punishment or delay (Denk et al., 2005; Cools et al., 2008; Schweighofer et al., 2008; Tanaka et al., 2009; den Ouden et al., 2013; Miyazaki et al., 2014), and extend to costs associated with the exertion of physical effort (Meyniel et al., 2016). Our finding confirms and expands this theory by demonstrating that enhancing central serotonin also modifies a subjective perception of cognitive effort costs in the context of information gathering. Here, we found that serotonin increased information gathering per se, independent of reward maximization. Additionally, computational modeling revealed that SSRI subjects were willing to pay small, local costs for more information, and this effect was particularly prominent in the costly condition. Critically, SSRIs did not impact choice response times, and it is therefore unlikely that serotonin affected the subjective cost of time in the task.

Subjective costs sit right at the heart of information gathering. Over recent years, a growing literature spanning nonhuman animal neurophysiology and computational modeling of human behavior demonstrates the relevance of subjective costs in the context of sequential sampling tasks (Thura and Cisek, 2016; Hauser et al., 2017b). In essence, mounting subjective costs means that subjects become less willing to sample more information as they go along, and are drawn toward making a decision in the absence of clear evidence. Neurophysiologically, it has been suggested this process may be implemented by a neural signal that increases over time, often termed urgency signal (Cisek et al., 2009; Yau et al., 2020). This urgency signal is added to an accumulation of evidence in (pre)motor areas, thus promoting decisions before absolute evidence is being gathered (Thura and Cisek, 2016).

In our computational model, we capture this behavioral signature by means of subjective sampling costs that are imposed on the action value for nondeciding, thus making a continuation of gathering novel information less and less likely. Replicating previous findings, we show such cognitive costs are low in the beginning but escalate in a nonlinear manner as sampling continues over time. This nonlinear emergence of costs can help explain suboptimalities in human behavior, with an undersampling in cost-free and an oversampling in costly environments (Bogacz et al., 2010; Hauser et al., 2017b).

Note that, in prior work, we found that greater information gathering, as revealed by a greater number of draws, e.g., in patients with OCD (Hauser et al., 2017a,b), or after noradrenergic neuromodulation (Hauser et al., 2018) can arise through a change in the indifference, or impatience, parameter that is governing after how many samples subjective costs start to escalate, i.e., the *p* parameter in our model. In contrast, however, in the current study, we found that serotonin specifically increased information gathering through a reduced sensitivity to the scaling of costs, i.e., the *cs* parameter of our model. This result underlines the strength of using computational models to analyze behavior as they can deliver a better mechanistic insight into cognitive processes, revealing effects that may otherwise be hidden to the experimenter.

In this study, we show that serotonin specifically reduces the scaling of such costs, meaning that subjective costs are impacting a decision less, regardless of how much one has already sampled. This finding has putative clinical relevance. SSRIs constitute a first-line intervention in the treatment of depression (Cipriani et al., 2018), a disorder typically characterized by motivational deficits, such as lowered willingness to engage in effortful behavior (Der-Avakian et al., 2016). Critically, using similar laboratory tasks, studies have shown that a willingness to gather information is reduced in patients suffering from depression (Taylor Tavares et al., 2007), as well as healthy individuals with low levels of motivation (Roets et al., 2008). Our study suggests that SSRI treatment can counteract such motivational deficits, through an increase in information gathering that is mediated by reduced cost perception. This result nicely extends previous computational studies that revealed how SSRI intervention increases physical effort exertion via reduced perception of effort costs (Meyniel et al., 2016). Taken together, these findings suggest that serotonin is not only relevant in signaling the costs of physical, but also of mental effort, hinting at a domain-general role of serotonin in overcoming aversive costs. Overall, this mechanism may help explain how serotonergic treatment gives rise to an alleviation of motivational deficits in patients suffering from depression.

More speculative is a link between our current finding and another psychiatric disorder, obsessive-compulsive disorder (OCD), that has been linked to aberrant information processing (Volans, 1976; Fear and Healy, 1997; Pélissier and O'Connor, 2002). Studies show (Hauser et al., 2017b; Voon et al., 2017), although not consistently (Chamberlain et al., 2007; Jacobsen et al., 2012), excessive information gathering behavior in OCD patients. Our findings suggest that SSRIs, first-line agents for treatment of OCD, could further exacerbate information gathering, although previous studies did not find an association with medication status (Hauser et al., 2017b). It is possible, however, that baseline serotonin levels may play a critical role in modulating an impact of serotonergic medication on information gathering. For instance, the relationship between serotonin levels and information gathering may follow an inverted u-shape function, which could also explain why a reduction of serotonin levels by means of tryptophan depletion in a previous study induced a change in sampling in a similar direction as the SSRI effects revealed in the current study (Crockett et al., 2012).

Clinically, serotonergic agents typically alleviate symptoms, such as low mood in depression, or obsessions in OCD, only after prolonged treatment of multiple weeks (Taylor et al., 2006; Fineberg et al., 2012). Interestingly, however, our results show that SSRIs modulate cognitive processes already in the first days of treatment. Notably, this chimes with prior studies on the effects of antidepressants on, e.g., emotional processing, learning and decision-making (Harmer and Cowen, 2013; Scholl et al., 2017; Michely et al., 2020, 2022), where these early effects are linked to later clinical treatment response (Tranter et al., 2009; Shiroma et al., 2014; Godlewska et al., 2016). Similarly, it is worth speculating that changes in sampling of information, as in our study, can, over time, lead to changes in symptoms in depression or OCD. Here, future studies in clinical populations over multiple weeks of treatment are needed to establish a link between these early cognitive effects and later clinical improvement and unravel the precise trajectory of this relationship.

In conclusion, our findings demonstrate that SSRI administration enhances information gathering. Computational modeling indicated this arises from a reduced perception of subjective sampling costs, rendering information gathering less cognitively effortful. Our findings point to a candidate mechanism by which serotonergic treatment might help alleviate motivational deficits in the context of a range of mental illnesses.
