# Supplementary material for: Boosting Serotonin Increases Information Gathering by Reducing Subjective Cognitive Costs
Source: J Neurosci. 2023 Aug 9;43(32):5848–55. doi: 10.1523/JNEUROSCI.1416-22.2023 (PMC10423044; doi:10.1523/JNEUROSCI.1416-22.2023)
Supplement: Extended Data Figure 1-1 — Self-report questionnaire data. We found no impact of drug on any of self-report questionnaires. BDI–II = Beck's Depression Inventory II (Beck et al., 1996); SHAPS = Snaith-Hamilton Pleasure Scale (Snaith et al., 1995); STAI = State-Trait Anxiety Inventory (Spielberger, 1983); PANAS = Positive and Negative Affective Scale (Watson et al., 1988). Download Figure 1-1, DOCX file. [file ns-JN-RM-1416-22-s01.docx]

|  | **placebo** |  | **citalopram** |  | **P_value_** |
| --- | --- | --- | --- | --- | --- |
|  |  |  |  |  |  |
| gender | 20 ♀ / 13 ♂ |  | 20 ♀ / 13 ♂ |  | 1.000 |
|  |  |  |  |  |  |
| age | 24.8 ± 3.9 |  | 24.5 ± 4.0 |  | 0.757 |
|  |  |  |  |  |  |
| BDI – II [day 1] | 4.4 ± 5.4 |  | 3.6 ± 4.0 |  | 0.540 |
| BDI – II [day 7] | 4.6 ± 5.7 |  | 4.5 ± 4.5 |  | 0.924 |
| BDI – II [day 7 – day 1] | 0.2 ± 3.3 |  | 0.8 ± 3.5 |  | 0.469 |
|  |  |  |  |  |  |
| SHAPS [day 1] | 0.3 ± 1.0 |  | 0.3 ± 0.7 |  | 1.000 |
| SHAPS [day 7] | 0.6 ± 1.6 |  | 0.8 ± 2.5 |  | 0.771 |
| SHAPS [day 7 – day 1] | 0.3 ± 1.5 |  | 0.5 ± 2.1 |  | 0.738 |
|  |  |  |  |  |  |
| STAI - state [day 1] | 30.6 ± 8.5 |  | 30.1 ± 6.4 |  | 0.795 |
| STAI - state [day 7] | 33.1 ± 9.7 |  | 31.4 ± 6.6 |  | 0.392 |
| STAI - state [day 7 – day 1] | 2.5 ± 8.5 |  | 1.4 ± 5.6 |  | 0.508 |
|  |  |  |  |  |  |
| STAI - trait [day 1] | 33.1 ± 9.7 |  | 34.6 ± 6.6 |  | 0.479 |
| STAI - trait [day 7] | 34.6 ± 9.8 |  | 35.5 ± 7.5 |  | 0.664 |
| STAI - trait [day 7 – day 1] | 1.5 ± 5.0 |  | 0.9 ± 3.1 |  | 0.615 |
|  |  |  |  |  |  |
| PANAS - positive [day 1] | 31.2 ± 8.6 |  | 30.0 ± 7.9 |  | 0.562 |
| PANAS - positive [day 7] | 29.0 ± 10.4 |  | 28.3 ± 8.3 |  | 0.775 |
| PANAS - positive [day 7 – day 1] | -2.3 ± 7.3 |  | -1.7 ± 5.6 |  | 0.749 |
|  |  |  |  |  |  |
| PANAS - negative [day 1] | 11.5 ± 2.5 |  | 11.2 ± 1.4 |  | 0.588 |
| PANAS - negative [day 7] | 12.1 ± 3.3 |  | 11.1 ± 1.7 |  | 0.143 |
| PANAS - negative [day 7 – day 1] | 0.5 ± 3.2 |  | -0.2 ± 1.8 |  | 0.277 |

**Figure 1-1**. *Self-report questionnaire data.*

We found no impact of drug on any of self-report questionnaires. BDI – II = Beck’s Depression Inventory II (Beck et al., 1996), SHAPS = Snaith-Hamilton Pleasure Scale (Snaith et al., 1995), STAI = State-Trait Anxiety Inventory (Spielberger, 1983), PANAS = Positive and Negative Affective Scale (Watson et al., 1988).
